# A New Cixiid Fossil Genus of the Tribe Acrotiarini from Mid-Cretaceous Burmese Amber (Insecta, Hemiptera, Fulgoromorpha) †

**DOI:** 10.3390/insects13010102

**Published:** 2022-01-17

**Authors:** Menglin Wang, Feiyang Liang, Thierry Bourgoin

**Affiliations:** 1 Key Laboratory of Southwest China Wildlife Resources Conservation, Ministry of Education, China West Normal University, Nanchong 637009, China; 2 Key Laboratory of Economic Crops Genetic Improvement and Integrated Utilization, Hunan University of Science and Technology, Xiangtan 411201, China; feiyang_sco@foxmail.com; 3 Institut de Systématique, Evolution, Biodiversité, ISYEB-UMR 7205 MNHN-CNRS-Sorbonne Université-EPHE-Univ. Antilles, Muséum National d’Histoire Naturelle, CP 50, 57 rue Cuvier, 75005 Paris, France; thierry.bourgoin@mnhn.fr

**Keywords:** planthopper, Fulgoroidea, Cixiidae, mesozoic, Myanmar, new taxon

## Abstract

**Simple Summary:**

Many fossil planthoppers (Hemiptera: Fulgoromorpha) have been recently described from Burmese amber. Most belong to extinct families already well known or newly described, but few are related to existing families and for which these fossils will prove to be very useful for future molecular calibration analyzes. Here, we describe a new fossil genus of the extant Cixiidae family that we place into the recently described fossil tribe Acrotiarini. A new key to genera identification of Acrotiarini is proposed. Allowing the description for the first time of the male genitalia of Acrotiarini, this new fossil genus broadens the knowledge of the fossil tribe, and it underlines the already great diversity of the Cixiidae in the Cretaceous.

**Abstract:**

A new Burmese amber genus *Maculixius* **gen. nov.** with its type species *Maculixius jiewenae* **sp. nov.** is described in the planthopper family Cixiidae. This new genus is unique in Burmese Cixiidae by its forewing venation, with Pcu and A1 merging in the middle of clavus, the late bifurcation of ScP+R slightly after this level, and the early CuA forking well before this level. Although the head capsule is missing, it belongs to the recently described tribe Acrotiarini, based on the pentacarinated mesonotum and the distinctly arched RA on forewing with cell C1 wider submedially than apically. Morphological characteristics of Acrotiarini are discussed, and a key to identification of Acrotiarini genera is provided. The new taxon broadens the knowledge of the tribe, and it underlines the already great diversity of the family in the Cretaceous.

## 1. Introduction

In the Cretaceous, the Fulgoromorpha hemiptera taxa already form a very diverse and probably numerous group of obligate sap-sucking phytophagous insects, evidenced by the diversity of their eleven families already observed in the Myanmar ambers. Three belong to well-known extant families: Achilidae Stål, 1866 [1], Cixiidae Spinola, 1839 [2], and Derbidae Spinola, 1839 [3], and eight to extinct ones: Dorytocidae Emeljanov & Shcherbakov, 2018 [4,5], Inoderbidae Shcherbakov & Emeljanov, 2021 [6], Jubisentidae Zhang, Ren & Yao, 2019 [7], Katlasidae Luo, Jiang & Szwedo, 2020 [8], Mimarachnidae Shcherbakov, 2007 [9,10,11,12,13,14,15], Perforissidae Shcherbakov, 2007 [16,17,18], and Yetkhatidae Song, Szwedo & Bourgoin, 2019 [19], in addition to a bizarre nymph probably belonging to Neazoniidae Szwedo, 2007 [2,20]. Although this diversity at the family level remains to be confirmed by phylogenetic analyses which could lead to synonymizing several of them, it underlines that the diversity of planthoppers was already high during the Cretaceous period, prior to the Cretaceous–Paleogene extinction episode.

Among them, the family Cixiidae Spinola, 1839 is the largest family in Fulgoromorpha, existing at least since the Cretaceous [2]. It is currently divided into 19 tribes with nearly 250 genera and some 2500 species worldwide [21]. The fossil tribe Acrotiarini Bourgoin & Luo, 2021 was recently described in Cixiidae to include three fossil genera: *Acrotiara* Bourgoin & Luo, 2021 (type species: *Acrotiara multigranulata* Luo & Bourgoin, 2021), *Delphitiara* Bourgoin & Luo, 2021 (type species: *Delphitiara tibiocoronata* Luo & Bourgoin, 2021), and *Pentacarinus* Bourgoin & Luo, 2021 (type species: *Pentacarinus kachinensis* Luo & Bourgoin, 2021). This tribe is only known from Burmese amber and can be typically recognized by a five-carinated mesonotum, a double frontal carina extending along the frons vanishing close to the median ocelli, and the RA vein on forewing distinctly arched, making cell C1 wider submedially than apically.

In this study, a fourth genus, *Maculixius* **gen. nov.**, with a new species, *M. jiewenae* **sp. nov.**, from Burmese amber in Acrotiarini is described. The characteristics to separate all known genera in Acrotiarini and a key to their identification are provided and discussed. Particularly, the male genitalia is described for the first time in Acrotiarini.

## 2. Materials and Methods

The specimen was collected in Hukawng Valley, Kachin State, northern Myanmar. This Burmese amber is documented from the Cenomanian period of the mid-Cretaceous as 98.79 ± 0.62 Ma based on U–Pb dating of zircons [22]. The amber specimen was observed with an Olympus SZX7 microscope, photographed with a Leica M205FA stereoscopic microscope, and processed with the software Heliconfocus. Measurements were conducted using the software LAS X connected to the Leica M205FA stereoscopic microscope. Line drawings were drawn using Coreldraw 2019. Terminologies follow Bourgoin et al. [23] for the forewing venation, Luo et al. [2] for the hindwing venation, and Bourgoin [24] for the male genitalia. The metatibiotarsal formula (t-st)/sI/sII corresponds to the number of lateral teeth (t) on the metatibia, the number of apical spines (st) on the metatibia, the number of apical spines (sI) on metatarsomere I, and the number of apical spines (sII) on metatarsomere II.

## 3. Systematic Paleontology

Order: Hemiptera Linnaeus, 1758
Suborder: Fulgoromorpha Evans, 1946
Superfamily: Fulgoroidea Latreille, 1807
Family: Cixiidae Spinola, 1839
Subfamily: Cixiinae Spinola, 1839
Tribe: Acrotiarini Bourgoin & Luo, 2021

**Genus: *Maculixius* Bourgoin et Wang, gen. nov**.

Type species: *Maculixius jiewenae* Wang et Bourgoin, sp. nov.
LSID urn:lsid:zoobank.org:act:C206EB68-FDFF-411D-ADDA-A24D94EE2939

**Diagnosis:** This new genus is unique in Acrotiarini by the following combined characters on forewing: RP with 3 terminals; Pcu and A1 fused in the middle of clavus; the fusion of Pcu and A1 earlier than the first bifurcation of ScP+R but later than the first bifurcation of CuA.

**Etymology:** The name is the combination of Latin word “*macula*” meaning spot, and the genus name “*Cixius*”, the type genus of the family Cixiidae, referring to the distinctive multi-spotted pattern on the forewing of the species. The gender is masculine.

**Description:**

**Figure 1:**
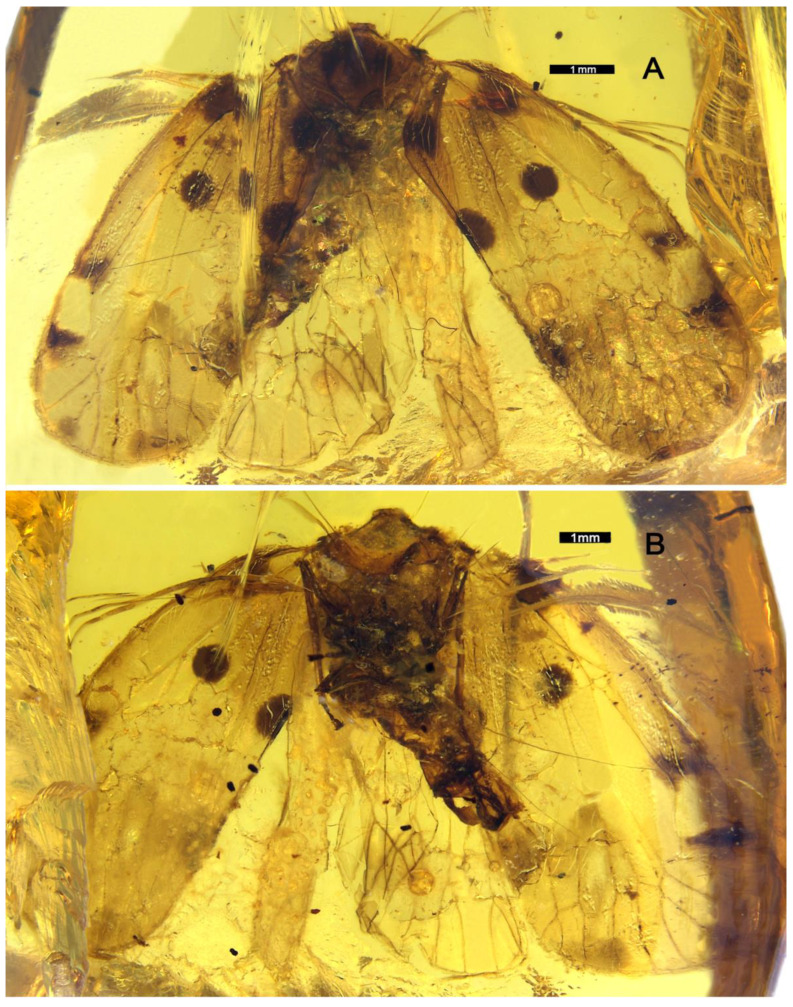
*Maculixiusjiewenae***sp. nov.**, Holotype, MDHP11. (**A**) Habitus, dorsal view. (**B**) Habitus, ventral view.

Condition of head, prothorax, and forelegs unknown (specimen damaged) (Figure 1)**.**

**Figure 2:**
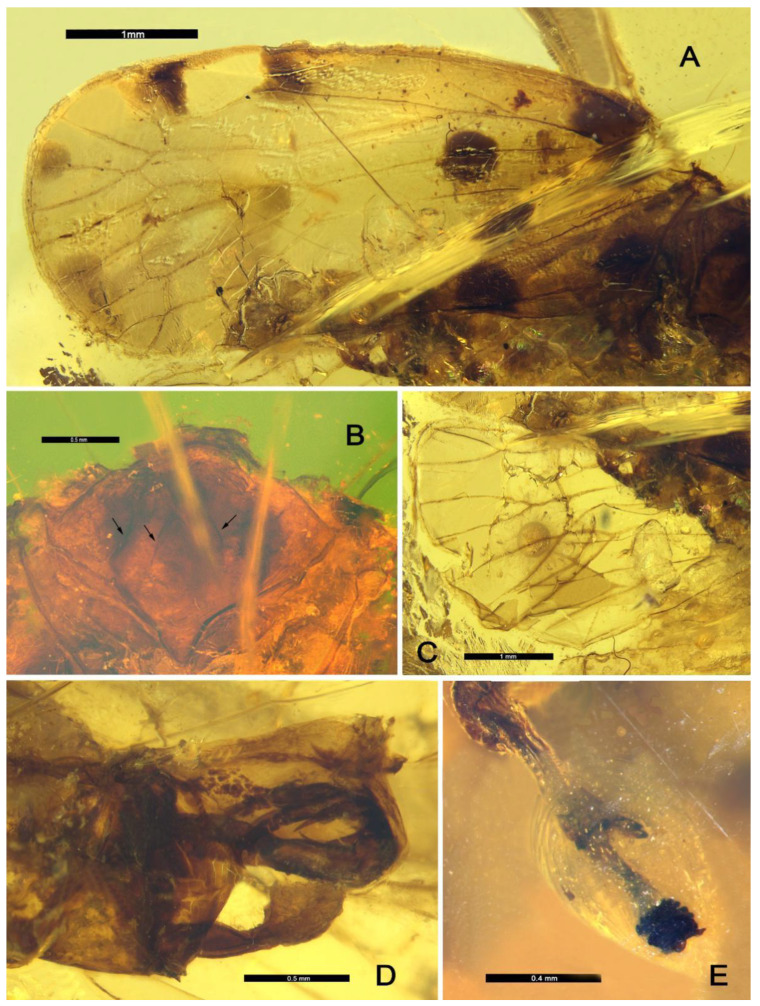
*Maculixius jiewenae***sp. nov.**, Holotype, MDHP11. (**A**) Left forewing, dorsal view. (**B**) Mesonotum, dorsal view. (**C**) Hindwing, dorsal view. (**D**) Male terminalia, lateral view. (**E**) Apex of tibia and tarsus on left leg, ventral view. The arrows indicate the visible carinae.

**Figure 3:**
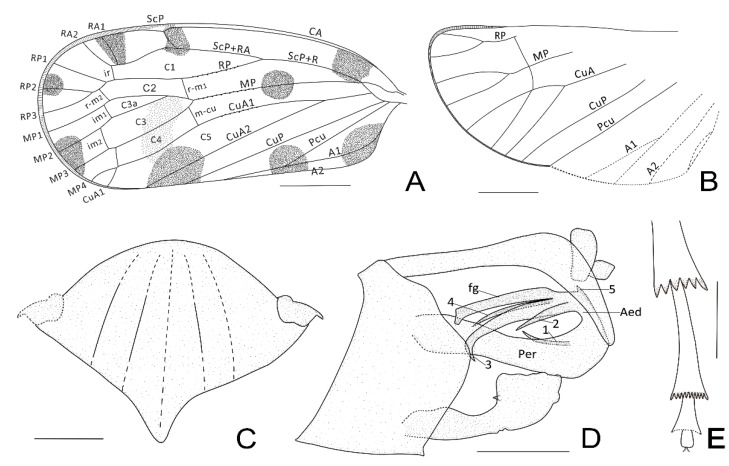
*Maculixius jiewenae***sp. nov.**, Holotype, MDHP11, line drawings. (**A**) Forewing schema. (**B**) Hindwing schema (claval part in dotted line difficult to observe). (**C**) Mesonotum, dorsal view. (**D**) Male terminalia, lateral view. (**E**) Apex of hind leg, lateral view (spines on tarsomere II in dotted line difficult to observe). Scale bars: 1 mm in (**A**,**B**), 0.5 mm in (**C**,**D**), 0.3 mm in (**E**). The abbreviations and numbers refer to the text.

**Thorax.** Mesonotum rhombus-shaped; anterior margin rounded; pentacarinated but median carina probably weak, poorly visible due to the condition of the fossil (Figure 2B and Figure 3C); tegula large (Figure 2B).

**Forewing.** More or less rectangular with anterior and posterior margins almost parallel (Figure 1A); posterior margin almost straight, not bent at the apex of clavus (Figure 1A); apical margin regularly rounded (Figure 1A); about 2.5 times as long as wide in midline; veins without visible setae but small setae insertions visible and regularly spaced on RP, MP, and CuA_1_ before the nodal line (Figure 2A and Figure 3A); peripheral membrane transversally wrinkled, vanishing after apex of clavus (Figure 2A and Figure 3A); pterostigma absent, peripherical membrane slightly widened at its level (Figure 2A and Figure 3A); clavus open, apex at about 2/3 of tegmen length (Figure 2A and Figure 3A); basal cell about 2.5 times as long as wide; ScP+R nearly parallel to costal margin, leaving basal cell in a common long stem and forking late in veins ScP+RA and RP at the basal 1/3 of tegmen after the level of Pcu and A1 fusion (Figure 3A); C1 long and wider at the nodal line level, distinctly anteriorly arched after forking of ScP and RA (Figure 3A); RA with 2 terminals (Figure 3A); RP reaching apical margin with 3 terminals (Figure 3A); *ir* between RA_2_ and unforked part of RP (Figure 3A); stem MP emerging from basal cell at almost the same point as ScP+R, firstly forked to MP_1+2_ and MP_3+4_ at nodal line level; MP_1+2_ short, forked again at apical 2/3 of tegmen, MP_1_ sinuate and MP_2_ nearly straight; MP_3+4_ long, forking at subapical line, MP_3_ simple, MP_4_ miming proximally a transverse vein touching CuA_1_ before running parallel to MP_3_ (Figure 3A); *rp-mp1+2* and *mp-cua1* at nodal line level (Figure 3A); transverse *ir*, distal *rp-mp1*, the 2 *im* and proximal part of MP_4_ more or less aligned in a subapical line (Figure 3A); stem CuA short, forked early at basal 1/4 of tegmen, well before ScP+R forking and Pcu-A1 merging levels; CuA_1_ long and slightly sinuate (Figure 3A); CuA_2_ simple and straight (Figure 3A); CuP straight, reaching apical 1/3 of tegmen; Pcu and A1 fused nearly half length of clavus (Figure 3A); Pcu+A1 stem reaching margin before CuP apex: clavus open (Figure 3A); cell C1 of acrotiarinian type (Figure 3A); C3 longer and wider than C2 (Figure 3A); C3a short, basally triangular (Figure 3A); C4 almost as long as C3 (Figure 3A); C5 probably closed after the nodal line by *cua1-cua2* but not visible on the fossil (Figure 3A).

**Hindwing**. Peripheral membrane transversally wrinkled, vanishing after apex of clavus (Figure 3B); RP with 2 terminals (Figure 2C and Figure 3B); MP with 3 terminals: MP_3+4_ single, meeting CuA_1_ punctually (X type) then separating again and meeting margin (Figure 3B); CuA with 2 terminals (Figure 3B); CuP, PCu, and A2 simple (Figure 3B); A1 probably forked (not observable in the fossil) (Figure 3B); margin very slightly notched at Pcu level (Figure 3B).

**Legs.** Middle leg slender, with femur probably flattened (Figure 1B); hind leg slender (Figure 1B); metatibia apparently without lateral spine, with a wide and strong row of 6 apical teeth.

**Male terminalia.** In lateral view, pygofer higher than wide, posterior margin sinuate, with a short medioventral process (Figure 3D); anal tube long with apical part curved ventrally, covering aedeagus apex (Figure 2D and Figure 3D); periandrium long, tubular with a distal spine-like process on left side (Figure 2D and Figure 3D); aedeagus asymmetric with a recurved dorsal flagellum bearing spine-like strong processes (Figure 2D and Figure 3D); gonostyli as long as pygofer in lateral view, ending in a more or less dorsally curved quadrangular formation and bearing a small spine-like process on its anterodorsal margin (Figure 2D and Figure 3D).

***Maculixius jiewenae* Wang et Bourgoin, sp. nov.**
LSID urn:lsid:zoobank.org:act:B85383AC-100D-43B1-A7CB-DD0EFF6F6FDF

**Etymology.** The new species is named after Mrs Dan Zuo’s daughter, Jiewen Zhao. As the donator of the type specimen, her mother hopes that this honor will promote Jiewen’s interests in natural history.

**Type specimen:** Holotype, MDHP11, deposited in the College of Life Science, China West Normal University, Sichuan, China.

**Type locality and age:** Hukawng Valley, Kachin State, northern Myanmar. Burmite amber from mid-Cretaceous Cenomanian period, 98.79 ± 0.62 Ma.

**Description:**

**Thorax.** Mesonotum 1.43 mm long in midline, 1.92 mm wide in widest part.

**Wings.** Tegmen 8.01 mm long from base to the apex, 3.28 mm wide in widest part; tegmen with 9 distinct blackish irregular large spots (Figure 1A, Figure 2A and Figure 3A): 4 roughly symmetrically distributed on each side of CuA at base of costal area and base of clavus, middle of clavus and mid-part of MP; 2 anteriorly to ScP vein and RA_1_ vein; 2 more, paler, along the apical margin, respectively on the end of RP_2_ and among the apex of MP_2_ and MP_3_; the 9th spot at apex of clavus continued with a fuscous band reaching MP anteriorly to nodal line.

**Legs.** Middle leg 1.30 mm long in femora and 2.34 mm long in tibia; hind leg with femora 1.10 mm long, tibia about 1.94 mm long; lateral metatibial spines absent, 6 apical metatibial spines tightly placed, of subequal length, diverging in a strong crown (Figure 2E and Figure 3E); tarsomere I with 11 apical spines (Figure 3E); apical spines of tarsomere II invisible due to the condition of the fossil; tarsal claws well developed (Figure 2E); metatibiotarsal formula: (0)-6/11/? (not observable).

**Male terminalia.** Pygofer in lateral view 1.00 mm high, 0.72 mm wide in its widest part, posterior margin with 2 convexities, the middle one larger than the one in upper 1/3 (Figure 2D and Figure 3D); anal tube long, arcuately decurved in apical 1/3 in lateral view (Figure 3D); periandrium long, slightly shorter than anal tube, tubular, bearing distally a thin sclerotized spine (1) curved upward on its dorsal left side (Figure 3D); aedeagus s.s. asymmetrical, with flagellum (fg) recurved dorsoanteriorly, more or less straight and unarmed, bearing several long and strong sclerotized spines pointing anteriorly: a shorter and thinner one (2) slightly basal on right side, a long one apically recurved downward one (3) passing on right side of the periandrium, a shorter one (4) placed on the left side of the periandrium, and probably a 5th short antero-dorsal one (5) at the base of the flagellum (Figure 3D); in lateral view, gonostyli medially thinner, dorsal margin bearing setae proximally, apically developed in a wide dorsal plate with its anterior margin bearing a short tooth-like process, dorsal margin almost straight, posteroventral margin widely convex (Figure 2D and Figure 3D).

## 4. Discussion

With a pentacarinated mesonotum, this new genus can be directly placed into the pentastirinian lineage [2] that groups the Pentastirini, Mnemosynini, and the Acrotiarini tribes. The tegmen, with the RA distinctly arched with cell C1 much wider submedially than apically, allows us to place this genus in Acrotiarini. Unfortunately, the loss of the head capsule prevents checking for the presence of the characteristics of paired submedian frontal carinae surrounding dorsally a slightly more elevated area and the straight frontoclypeal suture observed in the other Acrotiarini taxa. With other Acrotiarini genera, *Maculixius* shares the absence of a pterostigmal sclerotized plate; setiferous veins (although distinctly less dense in this new genus); the regular cixiid venation of the hindwing; the lack of lateral metatibial teeth and the distal ones strong, of subequal length; and absence of diastema. From the three other Acrotiarini genera already known, it differs by a plesio-morphic RP with 3 terminals, a late forking of stem ScP+R beyond the merging of Pcu+A1, and the more unusual early forking of CuA well before the level of Pcu and A1 merging on forewing.

A new identification key to Acrotiarini genera is provided accordingly:
1. RP with 3 terminals on forewing. Pcu and A1 merging in the middle of clavus. Stem ScP+R long, forking late after Pcu and A1 merging. CuA forking early, well before Pcu and A1 merging......*Maculixius* gen. nov.- RP with 2 terminals on forewing. Stem ScP+R short or of medium length......22. Pcu and A1 merging late at 2/3 of the clavus. Stem ScP+R short, forking early before CuA, both well before Pcu and A1 merging......*Pentacarinus* Bourgoin & Luo 2021- Pcu and A1 merging in the middle of clavus. ScP+R and CuA forking almost or slightly before the level of Pcu and A1 merging......33. Pedicel of antenna elongated-ovate. Two transverse veinlets distad to RA fork between RA and RP on forewing......*Delphitiara* Bourgoin & Luo, 2021- Pedicel of antenna short, conical. One transverse veinlet distad to RA fork between RA and RP on forewing......*Acrotiara* Bourgoin & Luo, 2021

*Maculixius* is the first male specimen known for the fossil tribe Acrotiarini. If, by chance, the specimen provides some details on the male genital structures for the tribe, the significant disparity and diversity of these structures existing in the current Pentastirini species [25] prevents us from suggesting any of these characteristics as diagnostic for the tribe. It remains however important to note: a long and tubular anal tube produced ventrodistally in lateral view, a long and tubular periandrium, and a typical recurved aedeagus with a long tubular flagellum, flanked by long sclerotized spines.

## 5. Conclusions

Numerous planthopper fossils remain to be described from Burma ambers, particularly within Cixiidae, for which, unfortunately, a better classification system and a solid phylogenetic frame allowing interpretation of the evolution of the family, are still missing. The new genus described broadens the knowledge of the tribe and underlines the already great diversity of the Cixiidae in the Cretaceous while it also expands our knowledge about their disparity. While providing additional morphological characters of the male genitalia, described for the first time here, which could help to place the tribe with more certainty relative to extant lineages of Cixiidae, it is also an additional landmark for future fossil calibration dating in molecular approaches.

## Data Availability

No new data were created or analyzed in this study. Data sharing is not applicable to this article.
